# *N*′-(2-Hy­droxy-1,2-diphenyl­ethyl­idene)benzohydrazide

**DOI:** 10.1107/S1600536810038365

**Published:** 2010-09-30

**Authors:** Ming-zhi Song

**Affiliations:** a College of Chemistry and Chemical Technology, Binzhou University, Binzhou 256600, Shandong, People’s Republic of China

## Abstract

In the title compound, C_21_H_18_N_2_O_2_, the amino group is involved in an intra­molecular N—H⋯O hydrogen bond. The rings make dihedral angles of 37.9 (2), 64.4 (2) and 83.6 (2)°. In the crystal, inter­molecular O—H⋯N and O—H⋯O hydrogen bonds link the mol­ecules into chains running along [100].

## Related literature

For related structures, see: Fun *et al.*(2008 ▶); Nie (2008 ▶); Seijas *et al.* (2007 ▶). For general background to the biological activity of Schiff bases and their metal complexes, see: Chakraborty *et al.* (1996 ▶); Jeewoth *et al.* (1999 ▶).
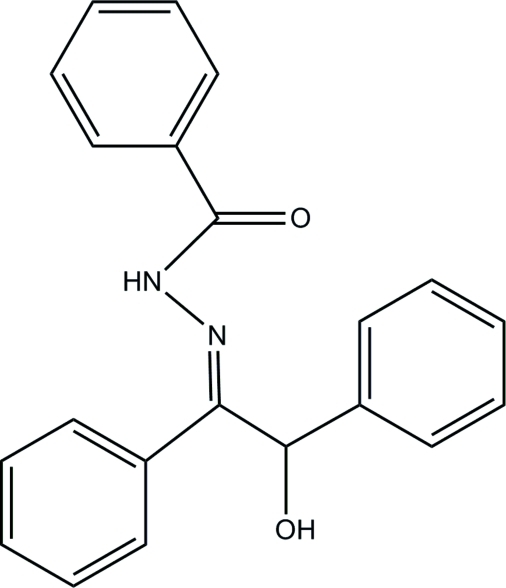


## Experimental

### 

#### Crystal data

C_21_H_18_N_2_O_2_
*M* *_r_* = 330.37
Orthorhombic, 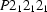

*a* = 7.7318 (8) Å
*b* = 11.0653 (12) Å
*c* = 19.725 (2) Å
*V* = 1687.5 (3) Å^3^
*Z* = 4
Mo *K*α radiation
μ = 0.09 mm^−1^
*T* = 298 K
0.41 × 0.16 × 0.13 mm

#### Data collection

Bruker SMART APEX CCD area-detector diffractometer
Absorption correction: multi-scan (*SADABS*; Sheldrick, 2007 ▶) *T*_min_ = 0.966, *T*_max_ = 0.989
7865 measured reflections
1729 independent reflections
1051 reflections with *I* > 2σ(*I*)
*R*_int_ = 0.054

#### Refinement

*R*[*F* ^2^ > 2σ(*F* ^2^)] = 0.040
*wR*(*F* ^2^) = 0.097
*S* = 1.07
1729 reflections
226 parameters
H-atom parameters constrained
Δρ_max_ = 0.14 e Å^−3^
Δρ_min_ = −0.16 e Å^−3^

### 

Data collection: *SMART* (Bruker, 2007 ▶); cell refinement: *SAINT* (Bruker, 2007 ▶); data reduction: *SAINT*; program(s) used to solve structure: *SHELXS97* (Sheldrick, 2008 ▶); program(s) used to refine structure: *SHELXL97* (Sheldrick, 2008 ▶); molecular graphics: *SHELXTL* (Sheldrick, 2008 ▶); software used to prepare material for publication: *SHELXTL*.

## Supplementary Material

Crystal structure: contains datablocks I, global. DOI: 10.1107/S1600536810038365/cv2764sup1.cif

Structure factors: contains datablocks I. DOI: 10.1107/S1600536810038365/cv2764Isup2.hkl

Additional supplementary materials:  crystallographic information; 3D view; checkCIF report

## Figures and Tables

**Table 1:** Hydrogen-bond geometry (Å, °)

*D*—H⋯*A*	*D*—H	H⋯*A*	*D*⋯*A*	*D*—H⋯*A*
O2—H2⋯O1^i^	0.82	2.45	2.998 (4)	125
O2—H2⋯N2^i^	0.82	2.20	2.992 (4)	164
N1—H1⋯O2	0.86	2.12	2.715 (4)	126

Symmetry code: (i) 
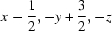
.

**Fig. 1.**
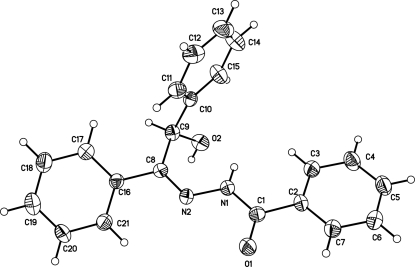
lThe molecular structure of (I) showing the atomic numbering scheme and 30% probability displacement ellipsoids.

## supplementary crystallographic information

### Comment

Schiff bases and their metal complexes were reported to exhibit fungicidal, bactericidal, antiviral, and antitubercular activity (Chakraborty *et al.*, 1996; Jeewoth *et al.*, 1999). Herewith we present the title compound (I), which is a new Shiff base compound.

In (I) (Fig. 1), the bond lengths and angles are normal and comparable to those observed in similar compounds (Nie *et al.*, 2008; Fun *et al.*, 2008) The C=N (C8=N2) bond length in the molecule is 1.287 (3) Å. Two benzene rings - C2—C7 and C16—C21, respectively - form a dihedral angle of  37.94 (7) °. The amino group is involved in formation of intramolecular N—H···O hydrogen bond (Table 1), while intermolecular O—H···N and O—H···O hydrogen bonds (Table 1) link the molecules into chains running in direction [100].

### Experimental

Benzoin (5 mmol), benzohydrazide (5 mmol) and methanol (10 ml) were mixed in 50 ml flask. After stirring for 2 h at 373 K, the resulting mixture was recrystalized from methanol, affording the title compound as a colorless crystalline solid. Elemental analysis: calculated for C_21_H_18_N_2_O_2_: C 76.34, H 5.49, N 8.48%; found: C 76.21, H 5.56, N 8.54%.

### Refinement

All H atoms were placed in geometrically idealized positions (N—H 0.86, O—H= 0.82 and C—H = 0.93–0.98 Å) and treated as riding on their parent atoms, with *U*_iso_(H) = 1.2U-1.5*U*_eq_ of the parent atom. Due to the absence of any significant anomalous scatterers in the molecule, the 1239 Friedel pairs were merged before the final refinement.

### Crystal data

**Table:**

C_21_H_18_N_2_O_2_	*D*_x_ = 1.300 Mg m^−^^3^
*M**_r_* = 330.37	Mo *K*α radiation, λ = 0.71073 Å
Orthorhombic, *P*2_1_2_1_2_1_	Cell parameters from 1315 reflections
*a* = 7.7318 (8) Å	θ = 2.8–19.7°
*b* = 11.0653 (12) Å	µ = 0.09 mm^−^^1^
*c* = 19.725 (2) Å	*T* = 298 K
*V* = 1687.5 (3) Å^3^	Needle, colourless
*Z* = 4	0.41 × 0.16 × 0.13 mm
*F*(000) = 696	

### Data collection

**Table:**

Bruker SMART APEX CCD area-detector diffractometer	1729 independent reflections
Radiation source: fine-focus sealed tube	1051 reflections with *I* > 2σ(*I*)
graphite	*R*_int_ = 0.054
phi and ω scans	θ_max_ = 25.0°, θ_min_ = 2.1°
Absorption correction: multi-scan (*SADABS*; Sheldrick, 2007)	*h* = −9→8
*T*_min_ = 0.966, *T*_max_ = 0.989	*k* = −13→13
7865 measured reflections	*l* = −10→23

### Refinement

**Table:**

Refinement on *F*^2^	Primary atom site location: structure-invariant direct methods
Least-squares matrix: full	Secondary atom site location: difference Fourier map
*R*[*F*^2^ > 2σ(*F*^2^)] = 0.040	Hydrogen site location: inferred from neighbouring sites
*wR*(*F*^2^) = 0.097	H-atom parameters constrained
*S* = 1.07	*w* = 1/[σ^2^(*F*_o_^2^) + (0.0315*P*)^2^ + 0.2666*P*] where *P* = (*F*_o_^2^ + 2*F*_c_^2^)/3
1729 reflections	(Δ/σ)_max_ < 0.001
226 parameters	Δρ_max_ = 0.14 e Å^−^^3^
0 restraints	Δρ_min_ = −0.16 e Å^−^^3^

### Special details

**Table:**

Geometry. All e.s.d.'s (except the e.s.d. in the dihedral angle between two l.s. planes) are estimated using the full covariance matrix. The cell e.s.d.'s are taken into account individually in the estimation of e.s.d.'s in distances, angles and torsion angles; correlations between e.s.d.'s in cell parameters are only used when they are defined by crystal symmetry. An approximate (isotropic) treatment of cell e.s.d.'s is used for estimating e.s.d.'s involving l.s. planes.
Refinement. Refinement of *F*^2^ against ALL reflections. The weighted *R*-factor *wR* and goodness of fit *S* are based on *F*^2^, conventional *R*-factors *R* are based on *F*, with *F* set to zero for negative *F*^2^. The threshold expression of *F*^2^ > σ(*F*^2^) is used only for calculating *R*-factors(gt) *etc*. and is not relevant to the choice of reflections for refinement. *R*-factors based on *F*^2^ are statistically about twice as large as those based on *F*, and *R*- factors based on ALL data will be even larger.

### Fractional atomic coordinates and isotropic or equivalent isotropic displacement parameters (Å^2^)

**Table:**

	*x*	*y*	*z*	*U*_iso_*/*U*_eq_	
N1	0.6095 (4)	0.7285 (3)	0.07224 (14)	0.0466 (9)	
H1	0.5711	0.6582	0.0833	0.056*	
N2	0.6267 (4)	0.7612 (3)	0.00542 (16)	0.0445 (9)	
O1	0.7060 (4)	0.9121 (2)	0.10546 (13)	0.0550 (8)	
O2	0.3956 (3)	0.5524 (2)	0.02563 (13)	0.0547 (8)	
H2	0.3161	0.5939	0.0110	0.082*	
C1	0.6551 (5)	0.8107 (4)	0.1207 (2)	0.0441 (10)	
C2	0.6408 (5)	0.7712 (3)	0.19238 (19)	0.0424 (10)	
C3	0.5837 (6)	0.6586 (4)	0.2127 (2)	0.0538 (12)	
H3	0.5485	0.6025	0.1805	0.065*	
C4	0.5787 (6)	0.6288 (4)	0.2806 (2)	0.0634 (13)	
H4	0.5385	0.5531	0.2939	0.076*	
C5	0.6324 (6)	0.7097 (4)	0.3285 (2)	0.0638 (13)	
H5	0.6309	0.6890	0.3742	0.077*	
C6	0.6885 (7)	0.8213 (4)	0.3087 (2)	0.0666 (14)	
H6	0.7230	0.8774	0.3410	0.080*	
C7	0.6939 (5)	0.8510 (4)	0.2408 (2)	0.0543 (11)	
H7	0.7344	0.9267	0.2279	0.065*	
C8	0.5919 (5)	0.6798 (3)	−0.03926 (18)	0.0416 (10)	
C9	0.5351 (5)	0.5513 (3)	−0.0227 (2)	0.0443 (10)	
H9	0.4936	0.5132	−0.0645	0.053*	
C10	0.6779 (5)	0.4743 (3)	0.00625 (18)	0.0404 (10)	
C11	0.8474 (5)	0.4869 (3)	−0.0146 (2)	0.0513 (11)	
H11	0.8759	0.5474	−0.0453	0.062*	
C12	0.9764 (6)	0.4104 (4)	0.0096 (2)	0.0650 (13)	
H12	1.0900	0.4197	−0.0050	0.078*	
C13	0.9345 (7)	0.3213 (4)	0.0551 (2)	0.0692 (14)	
H13	1.0199	0.2697	0.0714	0.083*	
C14	0.7678 (7)	0.3084 (4)	0.0766 (3)	0.0781 (15)	
H14	0.7401	0.2484	0.1077	0.094*	
C15	0.6399 (6)	0.3839 (4)	0.0523 (2)	0.0622 (12)	
H15	0.5267	0.3739	0.0672	0.075*	
C16	0.6030 (5)	0.7188 (3)	−0.11073 (19)	0.0414 (10)	
C17	0.6508 (6)	0.6403 (4)	−0.16168 (19)	0.0547 (11)	
H17	0.6767	0.5604	−0.1512	0.066*	
C18	0.6607 (6)	0.6793 (4)	−0.2285 (2)	0.0632 (13)	
H18	0.6950	0.6262	−0.2624	0.076*	
C19	0.6194 (6)	0.7971 (4)	−0.2443 (2)	0.0664 (14)	
H19	0.6252	0.8235	−0.2890	0.080*	
C20	0.5702 (6)	0.8749 (4)	−0.1946 (2)	0.0565 (12)	
H20	0.5415	0.9543	−0.2053	0.068*	
C21	0.5628 (5)	0.8365 (3)	−0.1283 (2)	0.0511 (11)	
H21	0.5301	0.8907	−0.0947	0.061*	

### Atomic displacement parameters (Å^2^)

**Table:**

	*U*^11^	*U*^22^	*U*^33^	*U*^12^	*U*^13^	*U*^23^
N1	0.062 (3)	0.0403 (18)	0.038 (2)	−0.0013 (18)	−0.0013 (18)	0.0009 (16)
N2	0.053 (2)	0.0448 (18)	0.0352 (18)	0.0040 (18)	0.0059 (17)	0.0001 (15)
O1	0.066 (2)	0.0506 (16)	0.0487 (17)	−0.0063 (15)	−0.0034 (16)	0.0039 (15)
O2	0.0445 (17)	0.0555 (16)	0.0642 (18)	−0.0006 (14)	0.0037 (16)	0.0091 (14)
C1	0.040 (2)	0.045 (3)	0.047 (3)	0.007 (2)	0.001 (2)	0.001 (2)
C2	0.040 (3)	0.050 (2)	0.037 (2)	0.001 (2)	0.000 (2)	0.005 (2)
C3	0.064 (3)	0.050 (3)	0.048 (3)	−0.004 (2)	−0.004 (2)	0.000 (2)
C4	0.074 (4)	0.064 (3)	0.052 (3)	−0.009 (3)	−0.001 (3)	0.015 (3)
C5	0.073 (4)	0.072 (3)	0.047 (3)	0.005 (3)	−0.001 (3)	0.012 (3)
C6	0.090 (4)	0.064 (3)	0.045 (3)	0.000 (3)	−0.003 (3)	−0.005 (2)
C7	0.060 (3)	0.054 (3)	0.050 (3)	−0.002 (2)	−0.005 (2)	0.000 (2)
C8	0.046 (3)	0.039 (2)	0.040 (2)	0.003 (2)	−0.001 (2)	−0.002 (2)
C9	0.043 (2)	0.047 (2)	0.043 (2)	−0.005 (2)	−0.002 (2)	0.000 (2)
C10	0.043 (2)	0.041 (2)	0.037 (2)	−0.0017 (19)	−0.006 (2)	−0.004 (2)
C11	0.051 (3)	0.045 (2)	0.057 (3)	−0.003 (2)	0.000 (3)	−0.002 (2)
C12	0.056 (3)	0.059 (3)	0.080 (3)	0.002 (3)	−0.012 (3)	−0.012 (3)
C13	0.067 (4)	0.063 (3)	0.078 (3)	0.015 (3)	−0.029 (3)	−0.003 (3)
C14	0.080 (4)	0.079 (4)	0.075 (3)	0.006 (3)	−0.013 (3)	0.028 (3)
C15	0.059 (3)	0.070 (3)	0.057 (3)	0.001 (3)	−0.002 (3)	0.018 (3)
C16	0.042 (3)	0.043 (2)	0.040 (2)	−0.001 (2)	0.004 (2)	0.000 (2)
C17	0.064 (3)	0.051 (2)	0.049 (2)	0.004 (3)	0.002 (2)	−0.002 (2)
C18	0.075 (3)	0.067 (3)	0.047 (3)	−0.014 (3)	0.007 (3)	−0.005 (3)
C19	0.070 (3)	0.084 (4)	0.045 (3)	−0.015 (3)	0.000 (3)	0.008 (3)
C20	0.064 (3)	0.056 (3)	0.049 (3)	−0.006 (3)	0.002 (2)	0.015 (2)
C21	0.057 (3)	0.049 (3)	0.047 (3)	−0.003 (2)	0.004 (2)	−0.003 (2)

### Geometric parameters (Å, °)

**Table:**

N1—C1	1.366 (4)	C10—C11	1.380 (5)
N1—N2	1.373 (4)	C10—C15	1.383 (5)
N1—H1	0.8600	C11—C12	1.392 (5)
N2—C8	1.288 (4)	C11—H11	0.9300
O1—C1	1.226 (4)	C12—C13	1.372 (6)
O2—C9	1.439 (4)	C12—H12	0.9300
O2—H2	0.8200	C13—C14	1.365 (7)
C1—C2	1.485 (5)	C13—H13	0.9300
C2—C7	1.365 (5)	C14—C15	1.379 (6)
C2—C3	1.381 (5)	C14—H14	0.9300
C3—C4	1.380 (5)	C15—H15	0.9300
C3—H3	0.9300	C16—C17	1.379 (5)
C4—C5	1.366 (5)	C16—C21	1.383 (5)
C4—H4	0.9300	C17—C18	1.389 (5)
C5—C6	1.366 (6)	C17—H17	0.9300
C5—H5	0.9300	C18—C19	1.378 (6)
C6—C7	1.378 (5)	C18—H18	0.9300
C6—H6	0.9300	C19—C20	1.359 (5)
C7—H7	0.9300	C19—H19	0.9300
C8—C16	1.477 (5)	C20—C21	1.375 (5)
C8—C9	1.524 (5)	C20—H20	0.9300
C9—C10	1.507 (5)	C21—H21	0.9300
C9—H9	0.9800		
			
C1—N1—N2	118.1 (3)	C11—C10—C9	121.7 (3)
C1—N1—H1	121.0	C15—C10—C9	120.1 (4)
N2—N1—H1	121.0	C10—C11—C12	121.1 (4)
C8—N2—N1	116.9 (3)	C10—C11—H11	119.4
C9—O2—H2	109.5	C12—C11—H11	119.4
O1—C1—N1	121.4 (4)	C13—C12—C11	119.4 (4)
O1—C1—C2	121.8 (4)	C13—C12—H12	120.3
N1—C1—C2	116.8 (4)	C11—C12—H12	120.3
C7—C2—C3	118.4 (4)	C14—C13—C12	120.1 (4)
C7—C2—C1	117.0 (3)	C14—C13—H13	120.0
C3—C2—C1	124.5 (4)	C12—C13—H13	120.0
C4—C3—C2	120.4 (4)	C13—C14—C15	120.4 (4)
C4—C3—H3	119.8	C13—C14—H14	119.8
C2—C3—H3	119.8	C15—C14—H14	119.8
C5—C4—C3	120.3 (4)	C14—C15—C10	120.9 (4)
C5—C4—H4	119.8	C14—C15—H15	119.6
C3—C4—H4	119.8	C10—C15—H15	119.6
C4—C5—C6	119.5 (4)	C17—C16—C21	118.1 (4)
C4—C5—H5	120.3	C17—C16—C8	121.8 (3)
C6—C5—H5	120.3	C21—C16—C8	120.1 (3)
C5—C6—C7	120.2 (4)	C16—C17—C18	120.7 (4)
C5—C6—H6	119.9	C16—C17—H17	119.7
C7—C6—H6	119.9	C18—C17—H17	119.7
C2—C7—C6	121.1 (4)	C19—C18—C17	119.7 (4)
C2—C7—H7	119.5	C19—C18—H18	120.1
C6—C7—H7	119.5	C17—C18—H18	120.1
N2—C8—C16	115.9 (3)	C20—C19—C18	120.1 (4)
N2—C8—C9	124.4 (3)	C20—C19—H19	120.0
C16—C8—C9	119.6 (3)	C18—C19—H19	120.0
O2—C9—C10	107.7 (3)	C19—C20—C21	120.1 (4)
O2—C9—C8	110.5 (3)	C19—C20—H20	120.0
C10—C9—C8	113.5 (3)	C21—C20—H20	120.0
O2—C9—H9	108.4	C20—C21—C16	121.3 (4)
C10—C9—H9	108.4	C20—C21—H21	119.3
C8—C9—H9	108.4	C16—C21—H21	119.3
C11—C10—C15	118.1 (4)		

### Hydrogen-bond geometry (Å, °)

**Table:**

*D*—H···*A*	*D*—H	H···*A*	*D*···*A*	*D*—H···*A*
O2—H2···O1^i^	0.82	2.45	2.998 (4)	125
O2—H2···N2^i^	0.82	2.20	2.992 (4)	164
N1—H1···O2	0.86	2.12	2.715 (4)	126

Symmetry codes: (i) *x*−1/2, −*y*+3/2, −*z*.
